# Thermal neutron beam optimization for PGNAA applications using Q-learning algorithm and neural network

**DOI:** 10.1038/s41598-022-12187-4

**Published:** 2022-05-23

**Authors:** Mona Zolfaghari, S. Farhad Masoudi, Faezeh Rahmani, Atefeh Fathi

**Affiliations:** Department of Physics, K.N. Toosi University of Technology, P.O. Box 15875-4416, Tehran, Iran

**Keywords:** Applied physics, Physics, Nuclear physics

## Abstract

As a powerful, non-destructive analysis tool based on thermal neutron capture reaction, prompt gamma neutron activation analysis (PGNAA) indeed requires the appropriate neutron source. Neutrons produced by electron Linac-based neutron sources should be thermalized to be appropriate for PGNAA. As a result, thermalization devices (TDs) are used for the usual fast neutron beam to simultaneously maximize the thermal neutron flux and minimize the non- thermal neutron flux at the beam port of TD. To achieve the desired thermal neutron flux, the optimized geometry of TD including the proper materials for moderators and collimator, as well as the optimized dimensions are required. In this context, TD optimization using only Monte Carlo approaches such as MCNP is a multi-parameter problem and time-consuming task. In this work, multilayer perceptron (MLP) neural network has been applied in combination with Q-learning algorithm to optimize the geometry of TD containing collimator and two moderators. Using MLP, both thickness and diameter of the collimator at the beam port of TD have first been optimized for different input electron energies of Linac as well as for moderators’ thickness values and the collimator. Then, the MLP has been learned by the thermal and non-thermal neutron flux simultaneously at the beam port of TD calculated by MCNPX2.6 code. After selecting the optimized geometry of the collimator, a combination of Q-learning algorithm and MLP artificial neural network have been used to find the optimal moderators’ thickness for different input electron energies of Linac. Results verify that the final optimum setup can be obtained based on the prepared dataset in a considerably smaller number of simulations compared to conventional calculation methods as implemented in MCNP.

## Introduction

Prompt gamma-ray neutron activation analysis (PGNAA) based on thermal neutron capture reaction is a fast, non-destructive, and accurate technique in multi-elemental neutron activation analysis, in which the gamma rays from activated nuclei of materials enable us to identify elements and determine their concentrations based on their energies and intensities, respectively^1–5^. The PGNAA technique, based on prompt gamma rays, is also an appropriate method for online measurement and exploration in manufacturing industries, petroleum and coal well-logging, landmines detection, and concealed explosives^6,7^.

Nuclear reactors, accelerators, ($\alpha ,\;n$) sources (e.g., ^241^Am/Be), spontaneous sources (e.g., ^252^Cf), and neutron generators (2.5 and 14.1 MeV neutrons from deuterium–deuterium (D–D) and deuterium–tritium (D–T) reactions, respectively) are general neutron sources which can be utilized in PGNAA^8–13^. In recent years, Linac-based photo-neutron sources are being used as an interesting alternative for providing neutron beams^14–16^.

However, neutron beams generated by such sources should be thermalized for PGNAA facility based on the thermal neutron capture reaction^17–21^. Thermalization devices (TDs) provide thermal neutron beams with minimum non-thermal neutron flux. A TD contains suitable materials for moderators and the collimator with optimized dimensions to achieve the desired thermal neutron beam. Since the properties of the obtained neutron beam depend on both materials and dimensions, TD optimization can accordingly be taken into account as a multi-parameter problem. Also, conventional computational codes such as MCNP^17–23^ are considerably time-consuming nevertheless; therefore, sequential optimization of dimensions in different parts of the TD is preferred, i.e., the characteristics of each layer (including appropriate materials and optimal dimensions) of TD against the neutron source should be selected through a large number of simulations. The next layers should be also optimized according to the properties of the previous layers. For example, the optimal TDs based on D-T neutron generator have been designed as a multi-parameter problem after the simulation of multiple programs using MCNP code for PGNAA^17,18^. Researchers also selected the appropriate materials and optimized the dimensions of two moderators and the collimator of the TD based on a 20 MeV electron Linac and spherical tungsten as electron photo-neutron converter using MCNPX code to achieve the maximum thermal neutron flux at the aperture of TD for PGNAA^21^.

In recent years, artificial intelligence (AI)-based methods such as artificial neural networks (ANN) and machine learning have been widely applied in many different areas, including nuclear physics for optimization purposes^24–30^.

In the present work, a hybrid method based on a combination of multilayer perceptron (MLP) artificial neural network and Q-learning algorithm has been proposed for optimizing four variables related to the dimensions of two moderators and the collimator of TD, as well as different electron energies of Linac. First, the optimal dimensions (thickness and diameter) of the collimator have been calculated using the MLP neural network, which was learned by 100 data of the thermal and non-thermal neutron flux simultaneously at the beam port of TD. To optimize the moderators’ thicknesses for different electron energies of Linac, the MLP neural network was then leaned by 300 data related to the properties of the thermal neutron at the beam port. Finally, the hybrid method has been applied which leads to a remarkable speed-up for optimizing TD dimensions in a more accurate way and with a smaller number of simulations compared to previous conventional methods.

## Materials and methods

**Figure 1:**
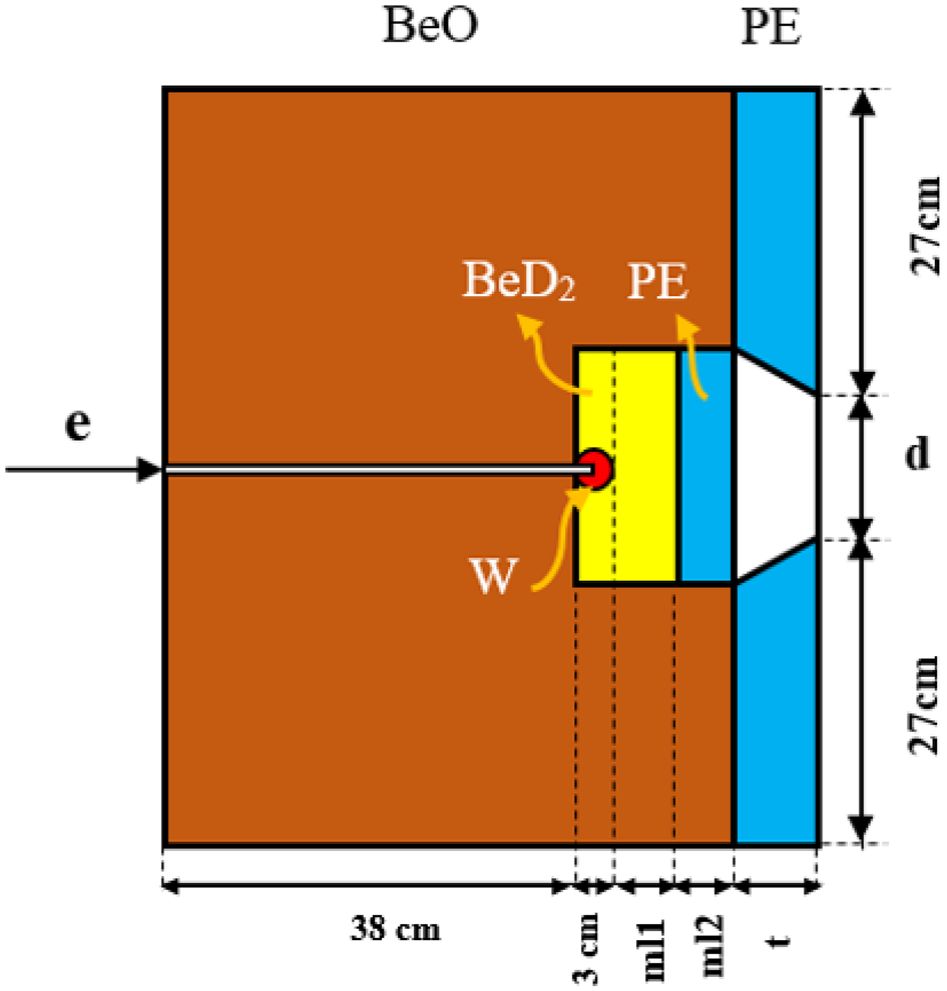
The primarily-proposed TD^21^.

For thermal neutron activation analysis purposes, it is required to consider the TDs surrounding a fast neutron source. In the design of the TD, an arrangement of materials including moderators, collimators, and reflectors with different dimensions has then been investigated using MCNPX2.6 code^17–23^. Following our recent work^21^, the best materials can be selected based on a smaller thermal neutron capture cross section (< 10^−2^ barn) among BeO, BeD_2_, Plexiglas, borated paraffin, carbon, polyethylene (PE), Teflon (CF_2_), and heavy water (D_2_O). To avoid design complexity (based on the fact that TD optimization is a multi-parameter problem), TD materials have been considered to be fixed^21^. We have previously shown that the maximum thermal neutron flux and the minimum non-thermal neutron flux in the neutron beam can be obtained simultaneously using two moderators (i.e., BeD_2_ and PE as the first and second moderators, respectively) with the optimized thickness of 4 cm in front of the spherical tungsten (electron converter and photo-neutron target)^21^. As shown in Fig. 1, the primarily-proposed TD is based on the photo-neutron target and an electron Linac including BeO (as the neutron reflector), BeD_2_ and PE (as the first and the second moderators, respectively) and PE (as the collimator). In addition, it should also be noted that the spherical tungsten as an optimized photo-neutron target with a radius of 1.5 cm produces higher fast neutron flux in comparison with other possible geometries^14^.

### Definition of thermalization efficiency

We aim at obtaining the optimal geometry of TD via simultaneous maximization of the thermal neutron flux $(\Phi_{th} )$, and minimization of the ratio of total to thermal neutron flux $(\Phi_{tot} /\Phi_{th} )$ in the neutron beam at TD’s beam port defined as thermalization efficiency (K)^17,18,21,23^. In the present work, K is a four-variable function for each Linac input electron energy as Eq. (1) indicates:

1 $$  {\text{K }}\left( {{\text{E}},{\text{ ml1}},{\text{ ml2}},{\text{ t}},{\text{ d}}} \right) \, = \left\{ {\Phi_{th} \left( {{\text{E}},{\text{ ml}}1,{\text{ ml}}2{ },{\text{ t}},{\text{ d}}} \right)} \right\}^{2} /\Phi_{tot} \left( {{\text{E}},{\text{ ml}}1,{\text{ ml}}2{ },{\text{ t}},{\text{ d}}} \right) \frac{n}{{cm^{2} \cdot mA}} $$

where, E is the Linac input electron energy, ml1 and ml2 are the thicknesses of the first (BeD_2_) and second (PE) moderators, and t and d are the thickness and diameter of the collimator (PE), respectively. Clearly, the larger K value at the beam port of the TD, the higher thermal neutron flux, which in turn leads to the smaller epithermal and fast neutron fluxes, and more efficient neutron beam for PGNAA applications as well.

To generate K as an MLP neural network dataset, the MCNPX2.6 code has been applied to transport electrons, photons, and neutrons using ENDF/B-VI Release 8 Photo-atomic Data (mcplib), as well as Photo-nuclear Data from ENDF7u libraries. In order for the results to be meaningful, all the simulations have been accordingly performed with relative errors less than 0.5%. The thermal, epithermal, and fast neutron energy bins for the F2 tally have also been considered for neutron flux calculation at the ranges < 10^–6^ MeV, 10^–6^–10^–2^ MeV, and > 10^–2^ MeV, respectively.

### Artificial neural network

ANN is a mathematical model inspired by human brain functionality. Neurons are the processing units in ANN with three main layers including input layer, hidden layer, and output layer. Each layer has several neurons connected to each other with synaptic weights. The sum of the weighted inputs is calculated in neurons to generate outputs affected by activation functions, which are usually nonlinear mathematical functions such as Tan-Sigmoid, Log-Sigmoid, and Rectifier as the commonly used activation functions^31,32^.

Mathematically, a neuron’s network function is governed by:

2 $$ {\text{Y}} = {\text{f}}\left( {\mathop \sum \limits_{i = 1}^{n} w_{i} x_{i} + b} \right) $$

where, Y is the output neuron, *x*_*i*_ is the input with the relative weight of *w*_*i*_, and b and f are the bias and activation functions, respectively.

According to the related information in each problem, the number of neurons in the input and output layers can be determined. The number of hidden layers depends on the nature of the problem. The number of neurons in the hidden layer can be estimated after many examinations. An MLP, as the most commonly-applied network, is a feedforward ANN that generally utilizes the back-propagation algorithm as the supervised learning method to train the network^31^. In the present study, the MLP neural network has been learned by 100 data of the K values at the first step of optimizing the dimension of the collimator for different electron energies and moderators’ thicknesses. After examinations (using python), the suitable configuration for the learned MLP neural network has been obtained with five input layers related to the associated five variables of the K function, seven hidden layers with a total of 1400 neurons, and one output layer.

At the second step of TD geometry optimization to find the optimal moderators’ thicknesses for different electron energies of Linac, the MLP neural network has been learned by 300 data of K values. The specifications of the trained MLP neural network model are three input layers due to the thicknesses of the two moderators for different input electron energies of Linac, three hidden layers with a total of 90 neurons, and one output layer. It should be mentioned that the Relu function has been utilized as the activation function of hidden and output layers at two steps.

To minimize the error between the network output or between predicted and the true K values during the training process, the weights connected to the neurons have been accordingly updated. The performance and quality of the produced MLP neural network model can also be estimated using mean squared error (MSE), mean absolute error (MAE), and R^2^-score as defined by Eqs. (3), (4), and (5)^32^. It should be noted that MSE is the average squared difference between the estimated and the true K values, MAE is the average of the absolute errors between the pair predicted and the true K values, and R^2^-score evaluates the performance of a linear regression model, defined between 0 to 1, as follows:

3 $$ {\text{MSE }} = \frac{1}{n} \mathop \sum \limits_{i = 1}^{n} \left( {Y_{i} - Y^{\prime}_{i} } \right)^{2} $$

4 $$ {\text{MAE }} = \frac{1}{n} \mathop \sum \limits_{i = 1}^{n} |Y_{i} - Y^{\prime}_{i} | $$

5 $$ {\text{R}}^{{2}}{\text{-Score }} = { 1} - \frac{{\mathop \sum \nolimits_{i = 1}^{n} \left( {Y_{i} - Y^{\prime}_{i} } \right)^{2} }}{{\mathop \sum \nolimits_{i = 1}^{n} \left( {Y_{i} - Y_{i}^{^{\prime\prime}} } \right)^{2} }} $$

where, $Y_{i} ,$ $Y^{\prime}_{i}$, and $Y_{i}^{{\prime\prime}}$ are the true value, the predicted value, and the mean of the true data, respectively, and n is also the number of data points.

### Reinforcement learning (RL) and Q-learning algorithm

**Figure 2:**
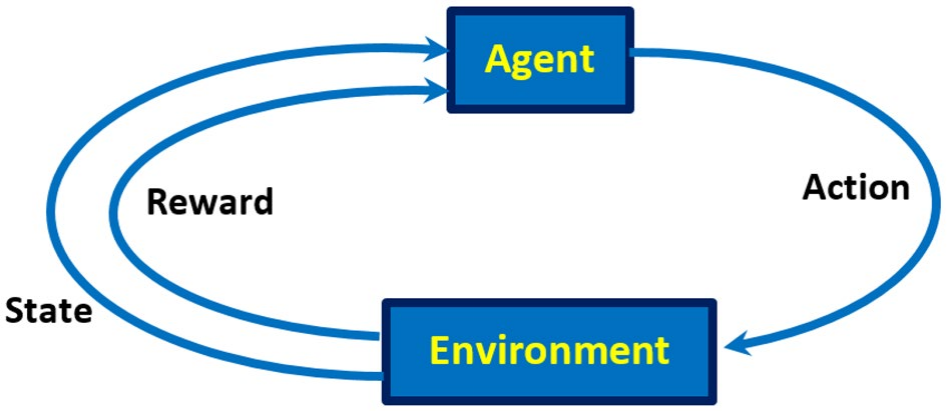
Agent’s performance in the environment.

RL is a part of machine learning that is not quite supervised or unsupervised. In principle, RL is related to human behavior, like a child learning to walk; therefore, RL is a trial-and-error learning approach. The RL includes agent, states, actions, environment, and receiving reward. The intelligent agent takes the best actions in an environment to maximize the cumulative reward. The agent’s performance in the environment is also shown in Fig. 2. In the present work, the agent is faced with four actions due to the increase and decrease of the thicknesses of the two moderators (BeD_2_ and PE as the first and the second moderator, respectively; forming also the states as well) within 1–10 cm with a 0.5-cm step to achieve maximum reward as K increases. The environment is also considered as a combination of these two values.

On the other hand, Q-learning is a value-based model of RL being applied to find the optimal cases in the problem using a Q-function. In the Q-learning algorithm, the Q-table is defined based on the rows of states and the columns of actions helping to calculate the maximum expected future rewards, as well as to select the best action at each state.

At the first step, the Q-table with *m* states (explained in the next section) in the rows and *n* (= 4) actions in the columns is initialized with the zero values.

At the second step, the agent randomly chooses and performs an action based on the epsilon greedy strategy.

Finally, the agent takes reward from the environment based on increasing K and updates the Q-function related to the new state. This three-step activity will be continued to complete the training of the agent and to exploit the environment entirely after the considered number (2000) of episodes in the Q-learning algorithm. The epsilon rate is a unit number defined for the random selection of actions in each episode. It should be noted that the epsilon rate is high in the beginning for the agent with no information about the environment. When the agent starts to explore the environment, however, the epsilon rate decreases, and then the agent can be able to exploit the combination of the two thickness values.

During the exploration process, the agent obtains more confidence to evaluate the Q values in the Q-table. The Bellman equation is utilized as the Q-function that takes two components of state (s), and action (a) which reads:

6 $$ newQ\left( {s,a} \right) = Q\left( {s,a} \right) + \alpha \left[ {R\left( {s,a} \right) + \gamma \max Q^{\prime}\left( {s^{\prime},a^{\prime}} \right) - Q\left( {s,a} \right)} \right] $$

where, Q(s, a) and newQ(s, a) are the Q values of the current state and new states, respectively. R(s, a) is the reward given by the environment at the current state considered which is 1.5 in this work, and $maxQ^{^{\prime}} \left( {s^{\prime}, a^{\prime}} \right)$ is the maximum future reward from the new state by selecting the best action in the current state. The $\alpha$ and γ values (both between 0 and 1) also indicate the learning rate and the discount factor, respectively. The learning rate is the step size of moving forward in each iteration by the agent. If it is closer to 1, the agent then considers only the new Q values; Consequently, it is preferred that the learning rate is 0.1 until the agent is learned in small-step sizes. The discount factor also indicates that the agent considers short-term and/or long-term rewards, so it has been considered to be 0.95 for long-term rewards^32,33^.

## Result and discussion

### Optimal dimensions of the collimator

At the first step in TD optimization, the thickness and diameter of the collimator have been investigated. For this purpose, 100 data of K values have been simulated using MCNPX2.6 code. Five variables of the K value have also been taken into account including: a 20 MeV input electron energy of Linac, two 4-cm thickness values for the first and second moderators, and different thickness and diameter values of the collimator within the ranges 1–10 cm and 6–15 cm (with a 1-cm step), respectively. Since the K values depend on the electron energies, normalized K values have been accordingly used for learning. 100 K values have been normalized to those related to the 5-cm thickness and 10-cm diameter of the collimator as the mean values of the two ranges aforementioned. According to our previous study^21^, the 4-cm thickness of BeD_2_ as the first moderator and that of PE as the second have been chosen as the optimized values based on a 20-MeV Linac and a 2-cm thickness-step using MCNP. Therefore, the range of thicknesses and diameter of the two moderators and collimator in current study that mentioned as a dataset for the MLP artificial neural network is based on our previous research results. The prepared dataset includes training data (56% of a dataset), validation data (14% of a dataset), and test data (30% of a dataset). The trained MLP model uses the training data; validation data has been applied to investigate the MLP neural network progress and to optimize the model; the test data has been also utilized to estimate the efficiency and performance of the MLP model. An epoch in the ANN further indicates one circuit in all training datasets^34^.

To achieve the proper configuration of the MLP neural network, and to extract the suitable training model, different hidden layers with different numbers of neurons, activation function types, optimizer types, and losses have been accordingly examined using python. As a result, the proper structure of the MLP neural network with seven hidden layers for this problem has been obtained. The specifications of the suggested MLP neural network are tabulated in Table 1.

**Table 1:** The specifications of the suggested MLP.

Network parameters	MLP
Number of inputs (x_i_)	5
Number of neurons in the first hidden layer	300
Number of neurons in the second hidden layer	200
Number of neurons in the third hidden layer	200
Number of neurons in the fourth hidden layer	200
Number of neurons in the fifth hidden layer	200
Number of neurons in the sixth hidden layer	200
Number of neurons in the seventh hidden layer	100
Number of outputs	1
Activation function of hidden and output layers	Relu function
Optimizer	Rmsprop = 0.001
Epoch	2500
Loss	0.001

**Figure 3:**
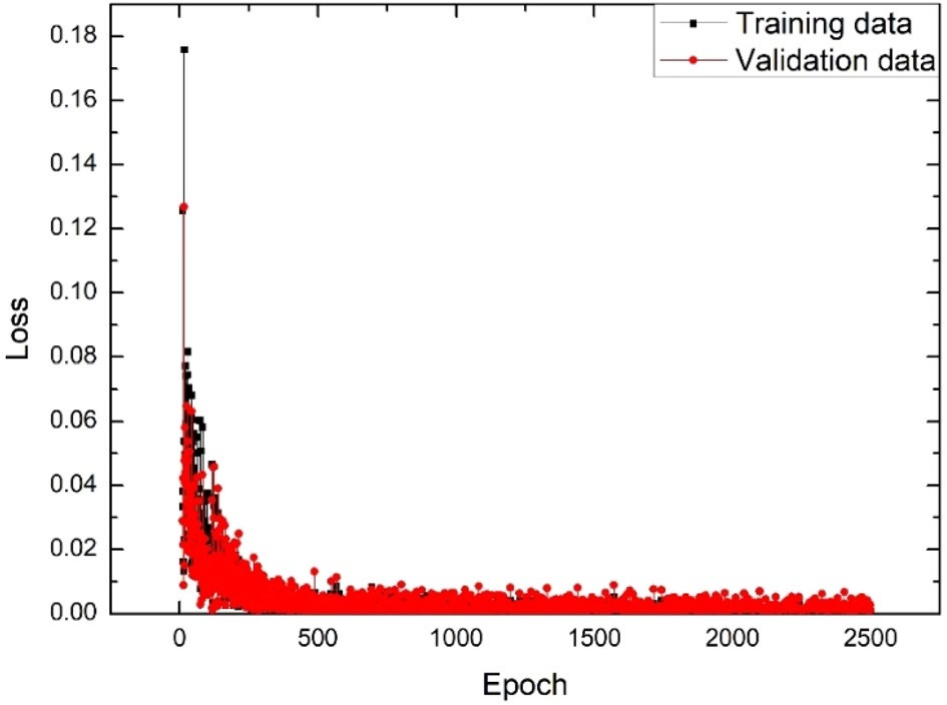
The loss for validation and training for 100 data in 2500 epochs.

To achieve the optimized weights of the MLP neural network, the difference between the real and predicted K values (namely, loss) should be minimized or become close to zero^35^. Accordingly, the loss for validation and training has been obtained for 100 data in 2500 epochs, as shown in Fig. 3.

**Figure 4:**
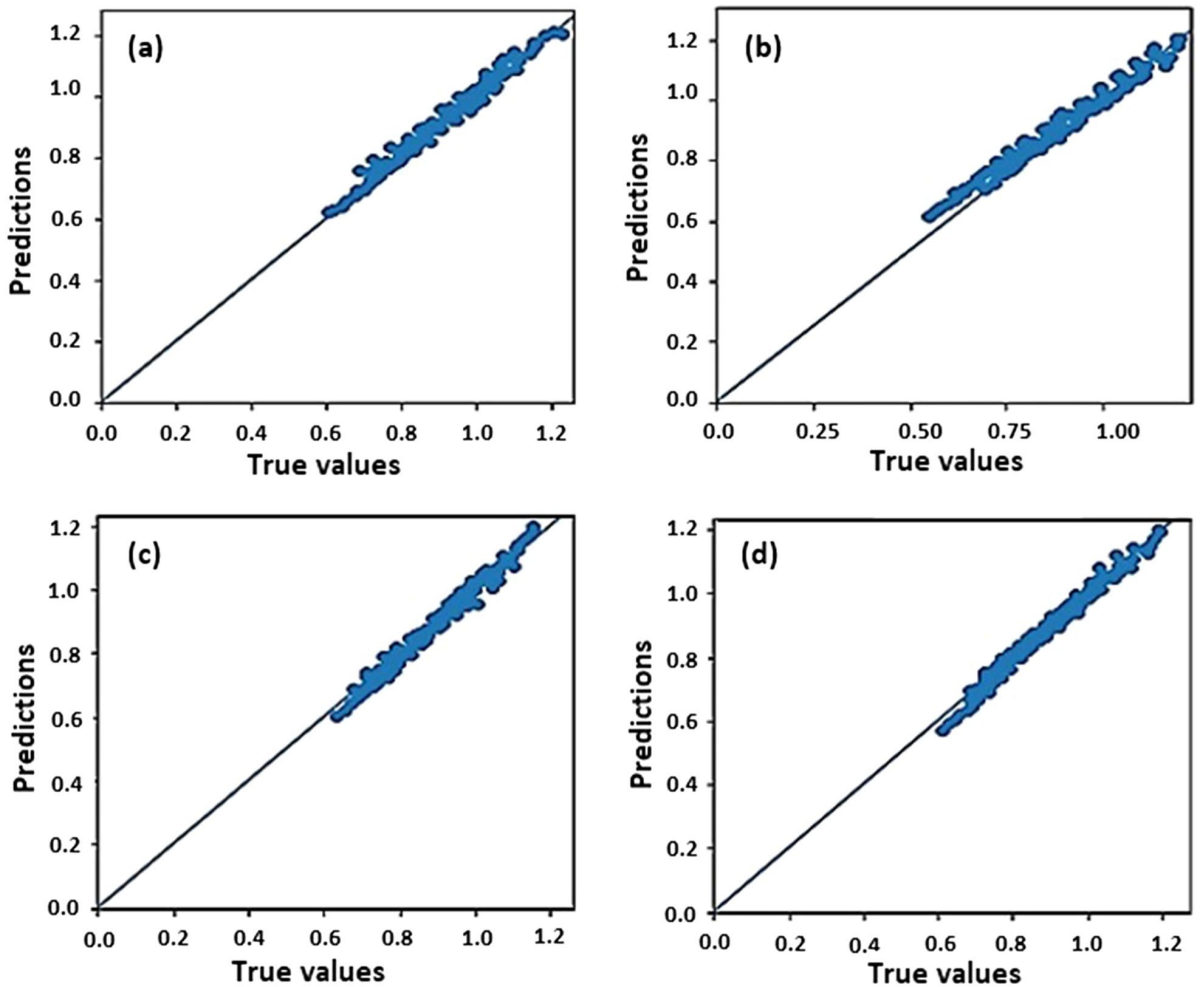
Testing the trained MLP neural network using the four sample datasets, each 100 data of K value with variables including (**a**) E = 18 MeV, ml1 = ml2 = 4 cm (**b**) E = 20 MeV, ml1 = 4 cm and ml2 = 3 cm, (**c**) E = 20 MeV ml1 = 4 cm and ml2 = 5 cm, (**d**) E = 25 MeV, ml1 = ml2 = 4 cm. Thickness and diameter values of the collimator are within the ranges 1–10 cm and 6–15 cm, respectively.

Also, the trained model extracted from the MLP neural network has been applied for testing and predicting the K values in the four sample datasets (Fig. 4). The variables associated to the K values in four datasets are: (a) E = 18 MeV, ml1 = 4 cm, ml2 = 4 cm; (b) E = 20 MeV, ml1 = 4 cm, ml2 = 3 cm; (c) E = 20 MeV, ml1 = 4 cm, ml2 = 5 cm; (d) E = 25 MeV, ml1 = 4 cm, ml2 = 4 cm, within the ranges 1–10 cm (for thickness value of the collimator) and 6–15 cm (for diameter value of the collimator) (100 data of K value in each dataset). The performance values of the trained MLP neural network represented by R^2^-score, MAE, and MSE are also tabulated in Table 2.

**Table 2:** Properties of the four sample datasets as well as the performance of the trained MLP model tested with them.

E^(1)^ (MeV)	ml1^(2)^ (cm)	ml2^(3)^ (cm)	R^2^-Score	MAE	MSE
18	4	4	0.97251	0.01858	0.00062
20	4	3	0.94709	0.03112	0.00148
20	4	5	0.97024	0.02012	0.00053
25	4	4	0.98041	0.01704	0.00042

^(1)^Input electron energy of Linac.

^(2)^Thickness of the first moderator (BeD_2_).

^(3)^Thickness of the second moderator (PE).

As seen in Fig. 3, the loss value for validation and training data approaches zero as epoch increases to 2500, meaning that the trained MLP neural network model has been optimized for predicting accurate K values for 2500 epochs.

From Fig. 4, in testing the trained MLP neural network with four datasets, both true and predicted K values are close to each other in the y = x line. As well, according to Table 2, the trained MLP neural network model has a high quality and performance for R^2^-score is close to 1, and for MAE and MSE to be nearly vanished.

Therefore, the MLP neural network model, trained with 100 data and tested with four sample datasets, can predict the normalized value of the thermalization efficiency. As a result, the maximum K value predicted by the trained MLP neural network model for different variables of the thermalization efficiency is achieved for 5 cm in thickness and 6 cm in diameter of the collimator. In other words, the optimized dimensions of the collimator are independent from both the electron energy and the thicknesses of the moderators. Hence, the 5-cm thickness and 6-cm diameter have been considered as the constant parameters throughout this work. Although the thermalization efficiency increases with the decrease in the diameter of the collimator (due to the smaller surface through which the neutron flux passes), according to the limitations of the sample size in NAA, a 6-cm diameter has been then considered as the minimum value for the collimator.

### Optimization of the moderators’ thickness values

**Figure 5:**
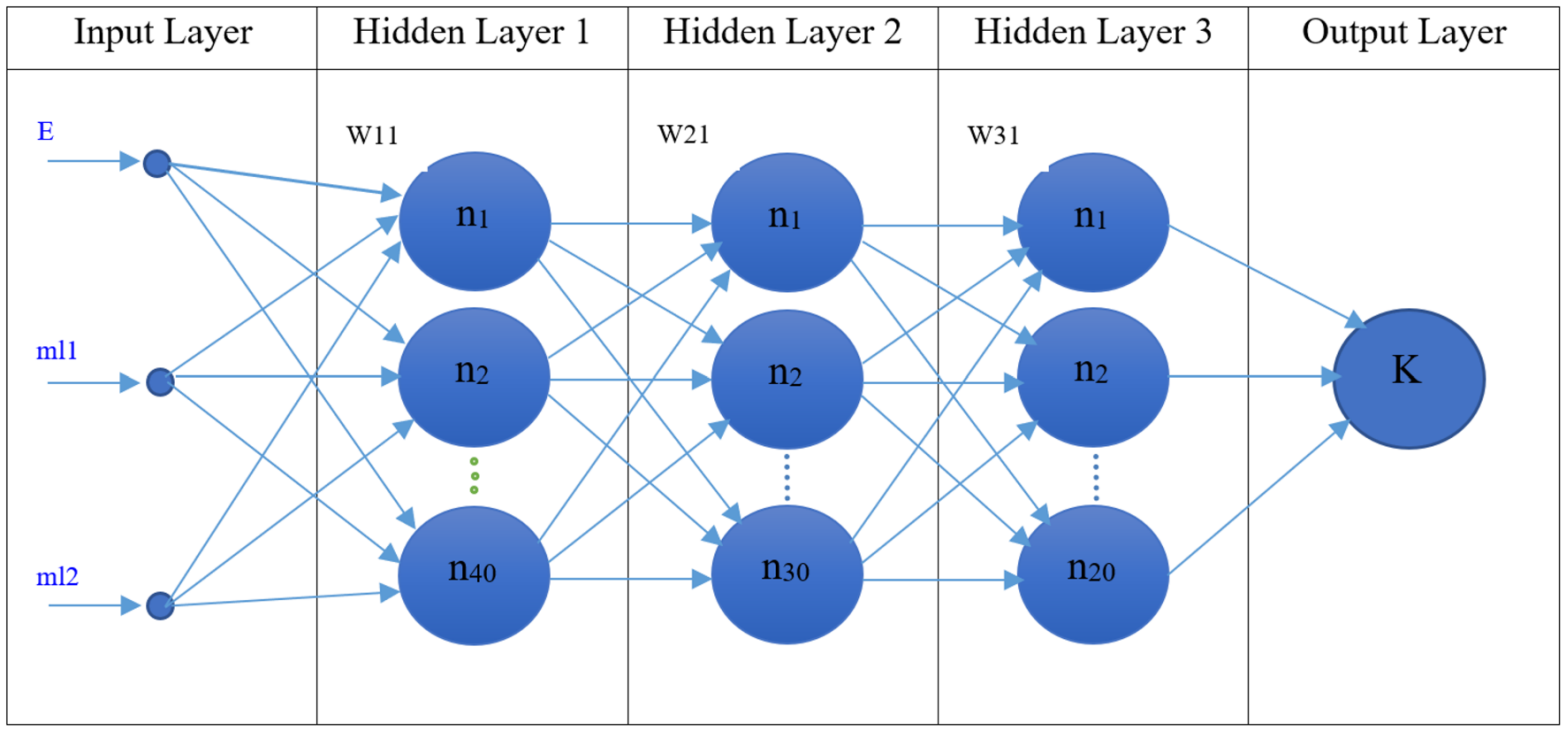
The appropriate configuration of the trained model of the MLP neural network for the 300 data of K value with three variables.

**Figure 6:**
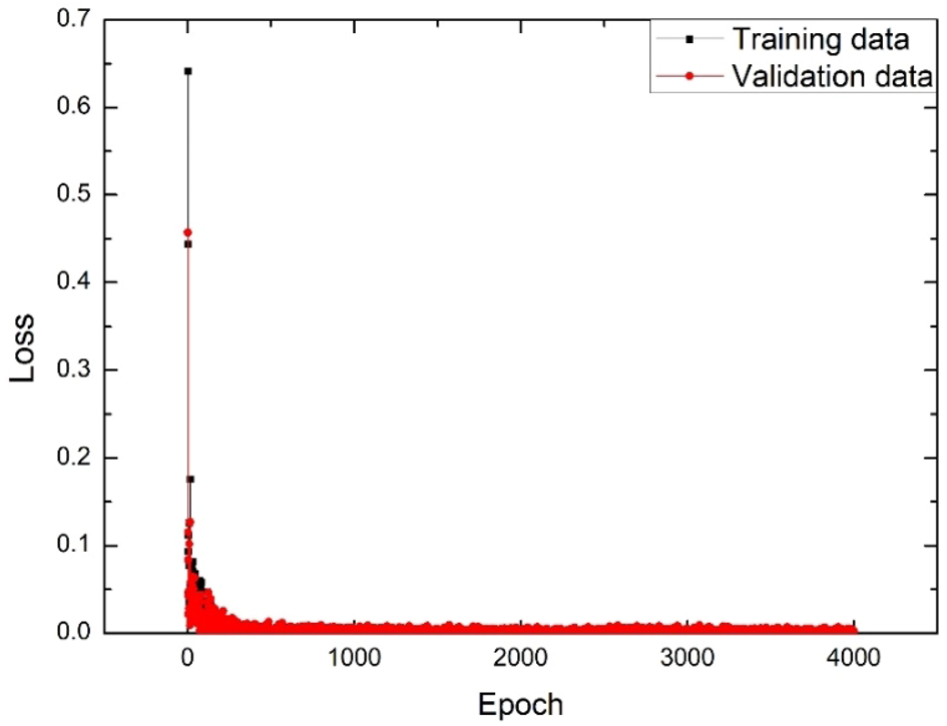
The loss for validation and training data for the 300 data of K value in 4000 epochs.

**Figure 7:**
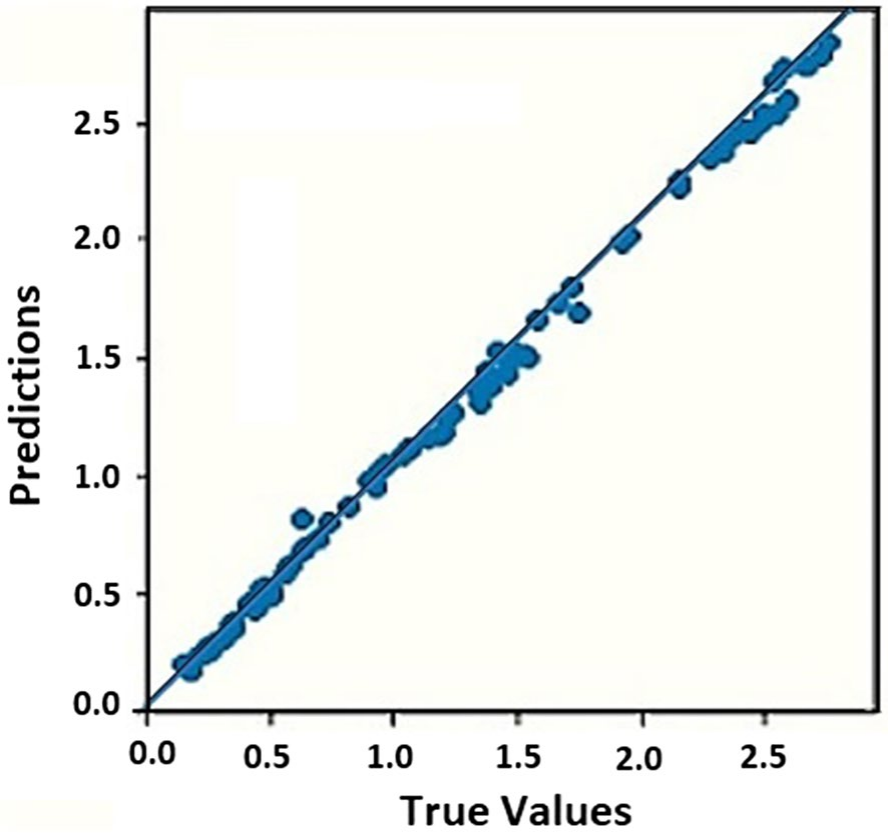
Testing the trained MLP neural network model with 30% of the 300 data of K value.

At the second step in TD optimization, the moderators’ thickness values for different input electron energies of Linac have been accordingly investigated. To determine the optimal thicknesses of BeD_2_ and PE respectively as the first and the second moderators, a combination of the Q-learning algorithm and MLP ANN has been then applied. The MLP neural network has been learned with the 300 data of K values with three variables; different input electron energies including 15, 20, and 25 MeV, and different thickness values for two moderators within 1–10 cm with a 1-cm step. Also, 56%, 14%, and 30% of the data have been considered for training, validation, and test data, respectively. After trial-and-error, the appropriate trained MLP neural network model has been obtained, with the configuration shown in Fig. 5, and with the specifications listed in Table 3. The loss for validation and training data has been also evaluated for the 300 data in 4000 epochs, as illustrated in Fig. 6. The trained model of the MLP neural network has been tested with 30% of the 300 data of K value, shown in Fig. 7. Also, the performance values of the learned MLP neural network have been tabulated in Table 4.

**Table 3:** Specifications of the trained MLP neural network model.

Network parameters	MLP
Number of inputs (x_i_)	3
Number of neurons in the first hidden layer	40
Number of neurons in the second hidden layer	30
Number of neurons in the third hidden layer	20
Number of outputs	1
Activation function of hidden and output layers	Relu function
Optimizer	Rmsprop = 0.8
Epoch	4000
Loss	0.0008

**Table 4:** The performance values of the learned MLP model tested with 30% of the 300 data of K value.

Epoch	R^2^-score	MAE	MSE
4000	0.98501	0.01604	0.00035

According to Fig. 6, validation and training data of the loss value for the 300 data are close to each other and drop to zero by increasing epochs up to 4000. Figure 7 shows the true and the predicted K values being approximately on the y = x line for the test data. Form Table 4, R^2^-score is also close to 1, while MAE and MSE are nearly vanishing. All of these values show that the trained MLP neural network model with the 300 data of K value based on different variables is optimized and also has a high performance in predicting K values for different variables of the K-function for 4000 epochs. Therefore, such a trained MLP neural network model can be utilized with the Q-learning algorithm to determine the optimal geometry of TD for different input electron energies of Linac. The purpose of using the Q-learning algorithm in this section is to find the optimal thickness values of BeD_2_ and PE as the first and second TD moderators simultaneously, within the range 1–10 cm (with a 0.5-cm step) in a short time and without running multiple simulations in comparison with MCNP. The combination of the Q-learning algorithm and the MLP neural network indeed enabled us to find the optimal thickness values of two moderators due to the optimal K value for different input electron energies of Linac. As a result, the training method indeed takes less effort due to the estimation of K values more accurately based on using both the trained model for different input electron energies, and more thickness values in few seconds. Preparing the database may be time-consuming, but every change in the next setups can be performed within few seconds; therefore, one needs to prepare the database only for one time and then every optimized setup can be designed in few seconds.

**Figure 8:**
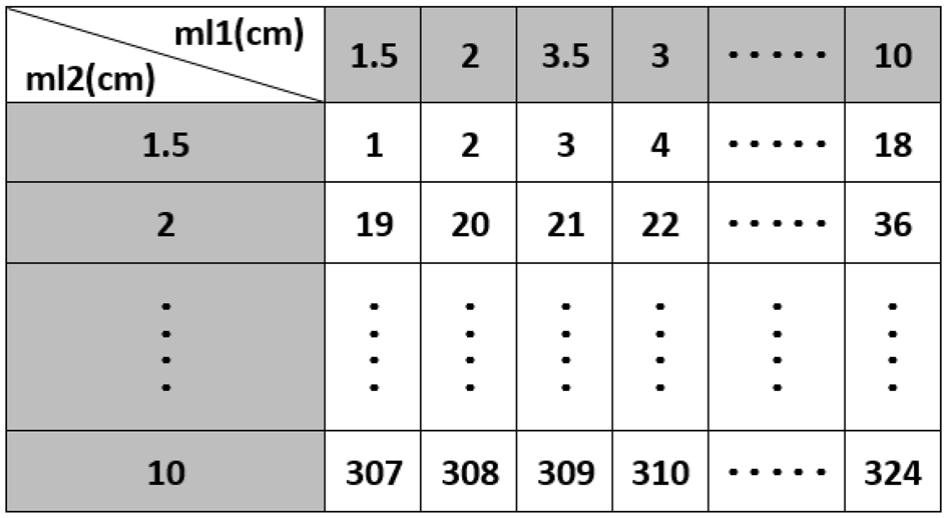
The 2D states table with 18 rows and 18 columns due to the second thickness of the moderator (PE) and the first thickness of the moderator (BeD_2_), respectively.

The Q-learning algorithm is based on the RL algorithm. Therefore, the RL parameters in this part are defined as follows: the combination of the thickness values of the two moderators as the environment, and those of the first and second moderators as the states, four actions related to the increase and decrease of the thickness values of these two moderators with a 0.5-cm step, and the received rewards based on increasing the K values. The states are related to the different thickness values of BeD_2_ and PE selected within 1–10 cm. The states table has been also defined in two dimensions with 18 rows and 18 columns (leading to 324 states) due to ml2 (moderator’s second thickness) and ml1 (moderator’s first thickness), respectively, as shown in Fig. 8. Thus, two actins (out of four) mention ml2 − 0.5 and ml2 + 0.5 cm due to change in PE thickness, and the other two mention ml1 + 0.5 and ml1 − 0.5 cm for change in BeD_2_ thickness_._ The ‘state-action’ pair during the Q-learning algorithm has also been defined in the Q-table.

**Figure 9:**
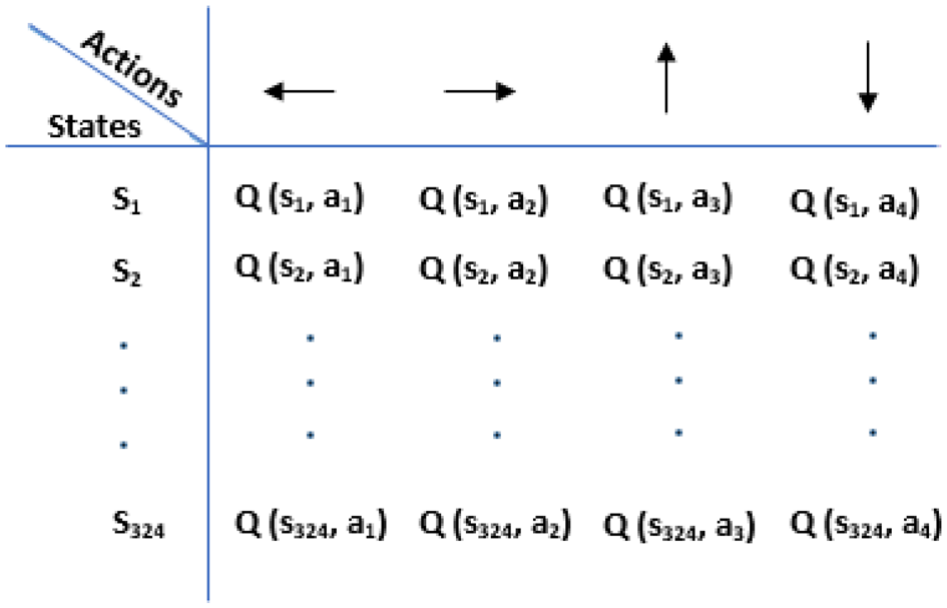
The Q-table with 324 states and 4 actions.

In this study, the Q-table includes 324 state rows and 4 action columns as shown in Fig. 9. Letting i be state number (or S_i_ in Q-table), number 1 (or S_1_) is associated to the 1.5-cm thickness values of both BeD_2_ (ml1) and PE (ml2), number 2 (or S_2_) is associated to the 2-cm thickness of BeD_2_ (ml1) and the 1.5-cm thickness of PE (ml2), and number 324 (or S_324_) is associated to the 10-cm thickness values of both BeD_2_ (ml1) and PE (ml2).

**Figure 10:**
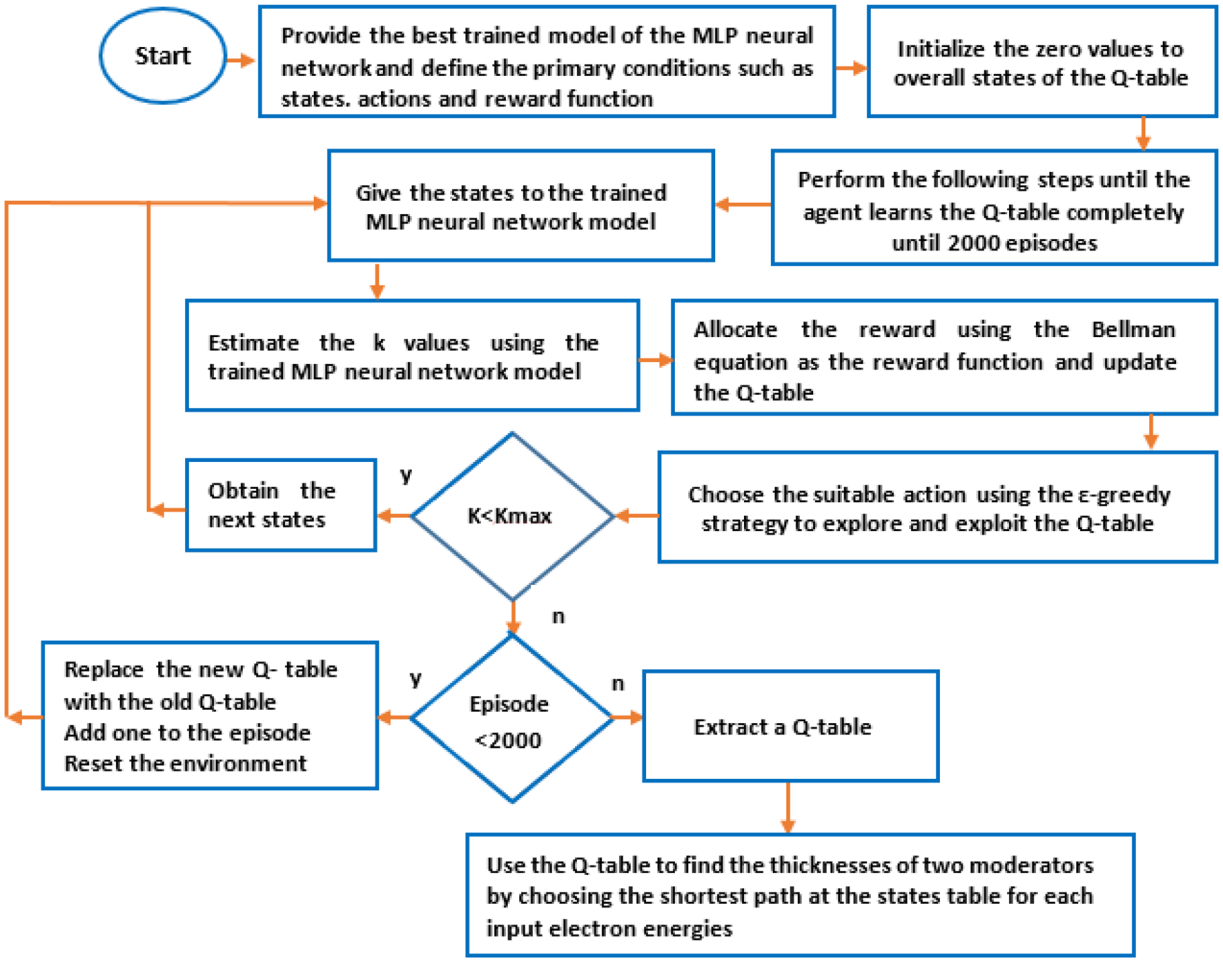
The flowchart of the performance of our hybrid Q-learning algorithm + the MLP neural network.

**Figure 11:**
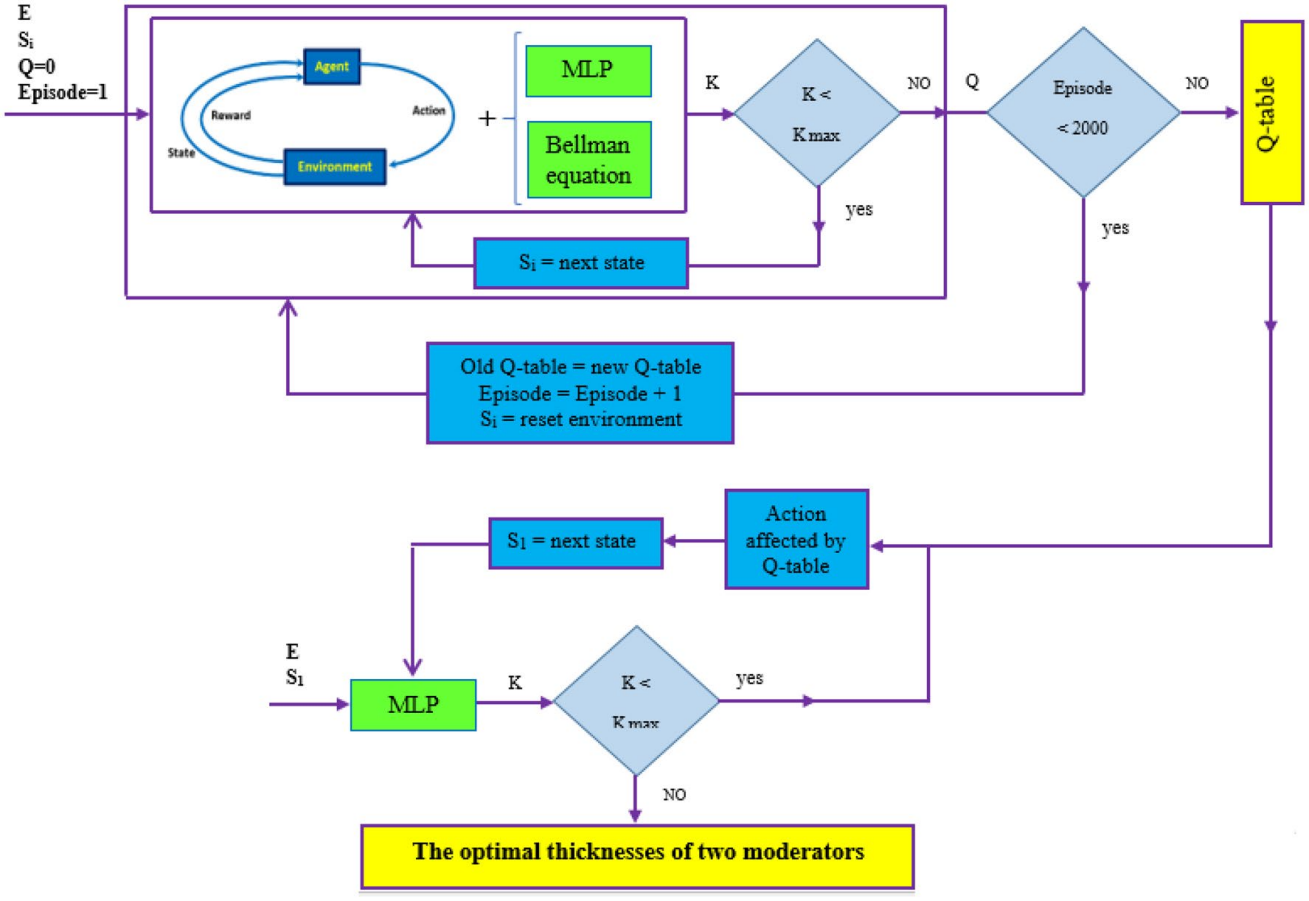
The flowchart of the performance of our hybrid Q-learning algorithm + the MLP neural network to obtain the optimal thickness values of the two moderators.

During the Q-learning algorithm, each ‘state-action’ pair of the Q-table initialized to zero is allocated to the value determined and updated by the Bellman equation (Eq. 6) with α = 0.1, γ = 0.95, and R (s, a) = 1.5 to obtain the maximum future rewards. As stated before, the action at a certain state has been selected randomly based on the epsilon greedy strategy to balance between exploration (the agent gets more accurate determining actions) and exploitation (the agent gets more rewards) to find the optimal thickness values of the two moderators. The probability of the epsilon greedy strategy has been chosen between 0 (exploitation) to 1 (exploration). In the beginning action selection and exploiting the environment, the epsilon greedy value has been set 1, meaning that the action will be selected completely in random. As the agent explores the environment, the epsilon then decreases by the decay rate of 0.01, while the probability of exploitation increases at each episode. The total number of episodes is also 2000. After exploiting the environment and extracting the Q-table, the actions will be affected by the Q values of the Q-table. Using the extracted Q-table and the MLP neural network, the agent takes both the initial thickness values of the two moderators and the input electron energy of Linac at the first step; and then moves in states table with a 0.5-cm thickness-step to reach the optimal thickness values and to obtain the maximum K value as well. Therefore, the combination of the Q-learning algorithm with the MLP neural network has been applied to find the optimal thickness values of the two moderators for different input electron energies of Linac and the related performances are schematically illustrated in Figs. 10 and 11. Also, as shown in Tables 5, 6, 7, the states and their paths to reach the optimal thickness values at the states table have been obtained by this hybrid method for some input electron energies of Linac (15, 20, and 25 MeV) in few seconds.

**Table 5:**
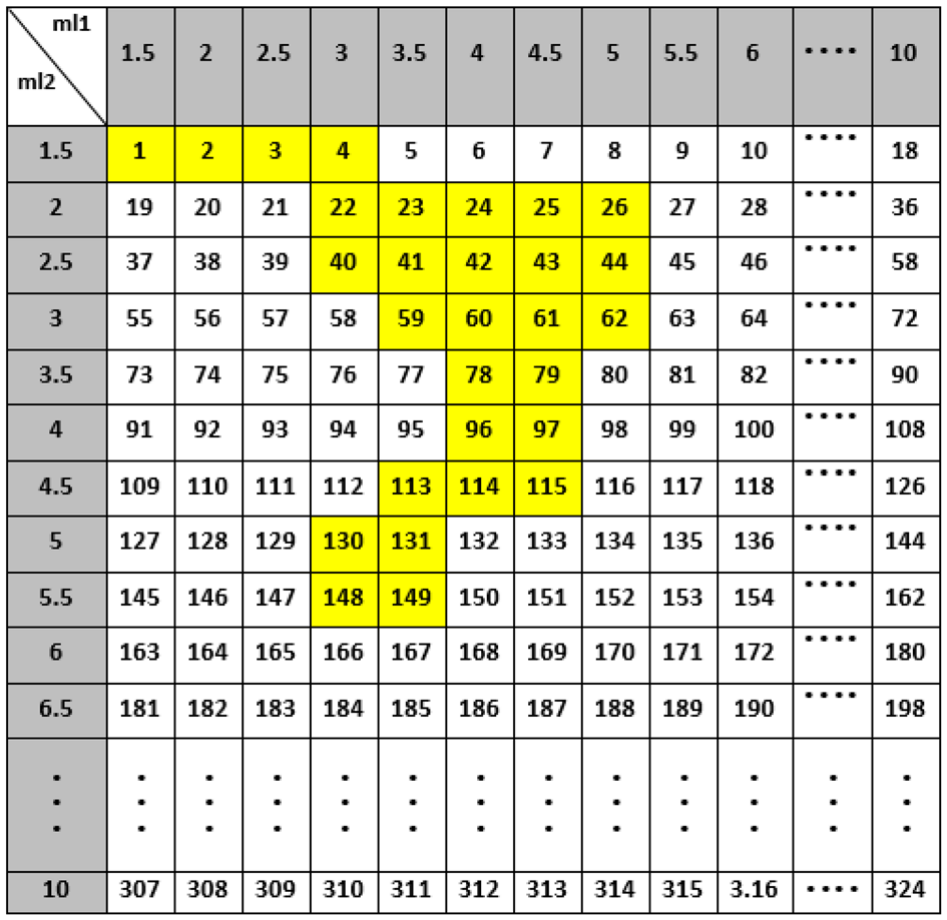
The shortest path of the moving agent to obtain the optimal thickness values of the two moderators for the 15 MeV input electron energy at the states table.

**Table 6:**
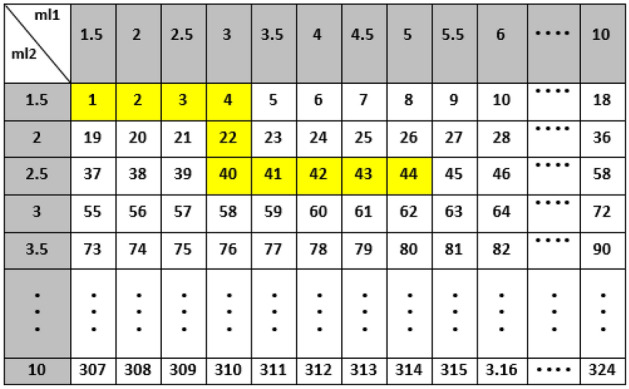
The shortest path of the moving agent to obtain the optimal thickness values of the two moderators for the 20-MeV input electron energy at the states table.

**Table 7:**
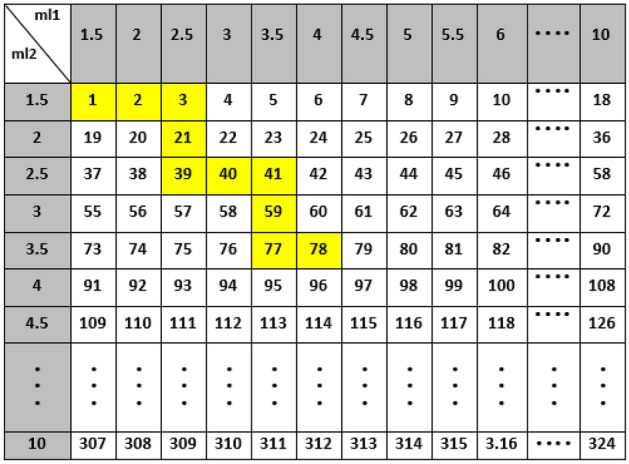
The shortest path of the moving agent to obtain the thickness values of the two moderators for the 25-MeV input electron energy at the states table.

According to Tables 5, 6, 7, the optimal thickness values of the first moderator and the second one indicating the optimal thermalization efficiency, are 4 and 3.5 cm for 25-MeV; 5.5 and 2.5 cm for 20-MeV; and 3.5 and 5.5 cm for 15-MeV input electron energies with K values of 2.77 × 10^10^, 1.57 × 10^10^ and 4.23 × 10^9^ (n/cm^2^ mA), respectively. Also, the highlighted values (in yellow) show the shortest paths and directions in which the agent moves in the states table with a 0.5-cm thickness-step to obtain the optimal thickness values of the two moderators for 15, 20, and 25 MeV input electron energies based on increasing the K values. The proposed hybrid method also enables us to recognize the least number of steps at the states table, which are 29, 11, and 10 for 15, 20, and 25 MeV, respectively, and show the faster speed of this innovative method compared to its conventional methods (e.g., MCNP). This method also enables us to estimate the optimal values of ml1 and ml2 simultaneously; therefore, in traditional method based on MCNP simulation, it is necessary to run separate codes for obtaining the optimal K value which is also time-consuming. In our hybrid method, the database is however prepared only for one time. For the next desired setups, the optimal K value is then determined within just few seconds. Investigating more thickness values within the range of 1–10 cm using fewer MCNPX codes is also another advantage of the proposed hybrid method. As a quantitative view, using only MCNPX to achieve the maximum K value at the states table requires the run of 324 codes (for one energy) for different thickness values of the two moderators within the range of 1–10 cm with a 0.5-cm thickness-step; therefore, 972 codes for 15, 20, and 25 MeV input electron energies, being considerably larger than 300 codes (in case of applying our hybrid method). Such a huge computational cost indeed requires very powerful computing hardware that considered as a drawback as well.

## Conclusion

The present study has been devoted to calculate TD’s optimal geometry for PGNAA applications using an accurate and rapid procedure. The optimal value of the thermalization efficiency related to the thickness and diameter of the collimator, as well as to the thickness values of the two moderators at the TD setup have been also estimated in two steps for different input electron energies including 15, 20, and 25 MeV. In the first step, the optimal dimensions of the collimator have been obtained using the trained MLP neural network with 100 data of K value. Then the moderators’ thickness values for different input electron energies of Linac have been optimized using the proposed hybrid method as a combination of MLP artificial neural network (trained with 300 data of K value) and the Q-learning algorithm. It has been found that the hybrid method is indeed capable of predicting K at different thickness values of the moderators ranging from 1 to 10 cm with a step of 0.5 cm for different input electron energies. The proposed method can also find the shortest path or the least number of steps to reach the optimal K-function for each input electron energy at the states table, enabling multi-parameter geometry optimization in a shorter period of time, and higher efficiency. It should be also noted that the applied trained model cannot be used for other TDs with different materials and geometries; however, the newly-prepared dataset and our proposed hybrid method can be used for similar problems.

## Data Availability

The datasets used and analyzed during the current study are available from the corresponding author on a reasonable request.
